# AGING, MIGRATION, AND GRANDPARENTING IN POST–ONE CHILD POLICY CHINA

**DOI:** 10.1093/geroni/igad104.3060

**Published:** 2023-12-21

**Authors:** Nan Zhang

**Affiliations:** University of Manchester, Manchester, England, United Kingdom

## Abstract

China’s rapid rural-urban migration and population ageing are posing significant challenges to traditional patterns of intergenerational familial support. To tackle population ageing, China’s one-child policy has been replaced by a two-child policy, subsequently a three-child policy. With limited institutional childcare facilities in place, ageing grandparents from rural areas often move to the cities where their adult migrant children settle to provide care for grandchildren. This group of older, rural people are largely marginalised and are invisible on the current research and policy agenda. This ongoing study, conducted in the Yangtze River delta urban area — one of the most developed, crowded, and the largest migrant-receiving urban regions in China — aims to uncover their experiences during the process of migration and adaptation, and establish the way in which this arrangement of grandparenting has impacted their everyday lives and well-being. Three compelling themes emerged at this stage: 1) intensified caring responsibilities imposed on older women largely owing to patriarchy; 2) intersection of old age, being women and exclusion of social welfare due to having a rural origin contribute to poor well-being; 3) the feeling of a lack of balance in terms of family exchange over the life course is not uncommon. It is possible that urbanisation will reinforce vulnerability and marginalisation of and exacerbate social exclusion of older women from rural origin. This project will form an important evidence base to tackle issues such as social exclusion of disadvantaged communities in China against the backdrop of rapid population ageing and urbanisation.

